# Audiovestibular Dysfunction in Alcohol Use Disorder: A Systematic Review of Human Primary Clinical Evidence

**DOI:** 10.3390/ijms27093905

**Published:** 2026-04-28

**Authors:** Jiann-Jy Chen, Chih-Wei Hsu, Brendon Stubbs, Tien-Yu Chen, Chih-Sung Liang, Yen-Wen Chen, Bing-Syuan Zeng, Ping-Tao Tseng

**Affiliations:** 1 Prospect Clinic for Otorhinolaryngology & Neurology, Kaohsiung 811, Taiwan; jiannjy@yahoo.com.tw (J.-J.C.); kevinachen0527@gmail.com (Y.-W.C.); 2 Department of Internal Medicine, E-Da Cancer Hospital, I-Shou University, Kaohsiung 824, Taiwan; 3 Department of Psychiatry, Kaohsiung Chang Gung Memorial Hospital and Chang Gung University College of Medicine, Kaohsiung 833, Taiwan; harwicacademia@gmail.com; 4 Psychological Medicine, Institute of Psychiatry, Psychology and Neuroscience (IoPPN), King’s College London, London WC2R 2LS, UK; brendon.stubbs@kcl.ac.uk; 5 Comprehensive Center for Clinical Neurosciences and Mental Health (C3NMH), Medical University of Vienna, 1090 Vienna, Austria; 6 Clinical Division of Social Psychiatry, Department of Psychiatry and Psychotherapy, Medical University of Vienna, 1090 Vienna, Austria; 7 Division of Psychology and Mental Health, Manchester Academic Health Science Centre, University of Manchester, Manchester M13 9PL, UK; 8 Department of Psychiatry, Tri-Service General Hospital, School of Medicine, National Defense Medical Center, Taipei 114, Taiwan; verducciwol@gmail.com; 9 Institute of Brain Science, National Yang Ming Chiao Tung University, Taipei 112, Taiwan; 10 Department of Psychiatry, Beitou Branch, Tri-Service General Hospital, School of Medicine, National Defense Medical Center, Taipei 112, Taiwan; lcsyfw@gmail.com; 11 Department of Psychiatry, National Defense Medical Center, Taipei 114, Taiwan; 12 School of Medicine, College of Medicine, National Sun Yat-sen University, Kaohsiung 804, Taiwan; 13 Institute of Precision Medicine, National Sun Yat-sen University, Kaohsiung 804, Taiwan

**Keywords:** alcohol use disorder, audiovestibular dysfunction, hearing loss, vestibular dysfunction, sensorineural hearing loss, thiamine

## Abstract

Alcohol use disorder is associated with substantial neurologic and systemic morbidity, but its relationship with audiovestibular dysfunction has not been clearly synthesized. This systematic review summarized human primary clinical evidence on auditory and vestibular manifestations, diagnostic findings, treatment, and prognosis in alcohol use disorder. PubMed, Embase, ClinicalKey, Web of Science, and ScienceDirect were searched from inception to 4 February 2026; the review followed PRISMA 2020 guidelines and was registered in PROSPERO (CRD420261301021). Because study designs, clinical contexts, and outcome measures were highly heterogeneous, a structured qualitative synthesis was performed. Twelve human primary clinical studies were included. The available evidence supported objective auditory dysfunction, most commonly sensorineural hearing impairment with frequent high-frequency involvement. Vestibular involvement was also reported, but the evidence was smaller and less phenotypically specific, consisting mainly of syndromic reports, broad peripheral/central classifications, and historical nystagmographic findings. Direct treatment evidence was very limited; improvement after thiamine replacement was reported in alcohol-related Wernicke-spectrum presentations, but no established disease-specific therapy was identified. Overall, current human clinical evidence supports heightened clinical awareness but not disease-specific screening algorithms or targeted therapeutic recommendations. Better prospective studies using contemporary phenotype-based audiovestibular assessment are needed.

## 1. Introduction

Alcohol has long been regarded as a potential ototoxic exposure capable of affecting both auditory and vestibular function [1]. Meta-analytic evidence has demonstrated an increased risk of hearing loss associated with alcohol consumption [2], and alcohol-related dizziness/vertigo has also been reported in other clinical contexts [3]. Epidemiological studies further indicate that individuals consuming two or more units of alcohol per day exhibit an increased risk of idiopathic sudden sensorineural hearing loss [4]. Among individuals with alcohol dependence, prevalence estimates of hearing impairment have ranged from 16.9% [5] to over 73.3% [6] in small clinical studies [7].

Despite many individuals reporting no subjective hearing difficulties related to alcohol consumption [8], objective assessments have repeatedly demonstrated alcohol-related audiovestibular abnormalities. Acute alcohol ingestion has been shown to elevate hearing thresholds in a dose-dependent manner, particularly at low frequencies critical for speech discrimination, with additional suppression of distortion product otoacoustic emissions at high frequencies [9,10]. Vestibular dysfunction has also been documented in individuals with chronic alcohol use, with clinical and electronystagmographic evidence suggesting central vestibular involvement in some studies [11].

The mechanisms linking alcohol use disorder to audiovestibular dysfunction are likely multifactorial. Thiamine deficiency and Wernicke encephalopathy have been extensively implicated, arising from inadequate intake, impaired absorption, and disrupted cellular utilisation of thiamine [12,13,14]. Thiamine deficiency promotes N-methyl-D-aspartate receptor-mediated excitotoxicity, resulting in characteristic lesions within thalamic, paraventricular, and periaqueductal regions that may disrupt central auditory and vestibular processing [15,16,17]. Nevertheless, conflicting evidence exists, with some studies reporting neutral or protective associations between moderate alcohol intake and hearing outcomes [18,19].

Importantly, most existing literature has addressed alcohol consumption in general rather than alcohol use disorder, a more severe and sustained condition associated with greater neurologic, metabolic, and nutritional burden [20,21]. In addition, the clinical literature specific to alcohol use disorder is scattered across small observational studies and descriptive reports, with substantial heterogeneity in outcome definitions, diagnostic methods, and clinical context [22]. This fragmentation has limited clear synthesis of the human clinical evidence and has contributed to uncertainty regarding the extent, pattern, diagnosis, and management of alcohol use disorder-related audiovestibular dysfunction. Accordingly, the present systematic review aimed to synthesize human primary clinical evidence on the auditory and vestibular manifestations, diagnostic findings, treatment, and prognosis of audiovestibular dysfunction in individuals with alcohol use disorder, in order to provide a more clinically grounded summary of the existing evidence and to identify priorities for future research.

## 2. Results

**Figure 1:**
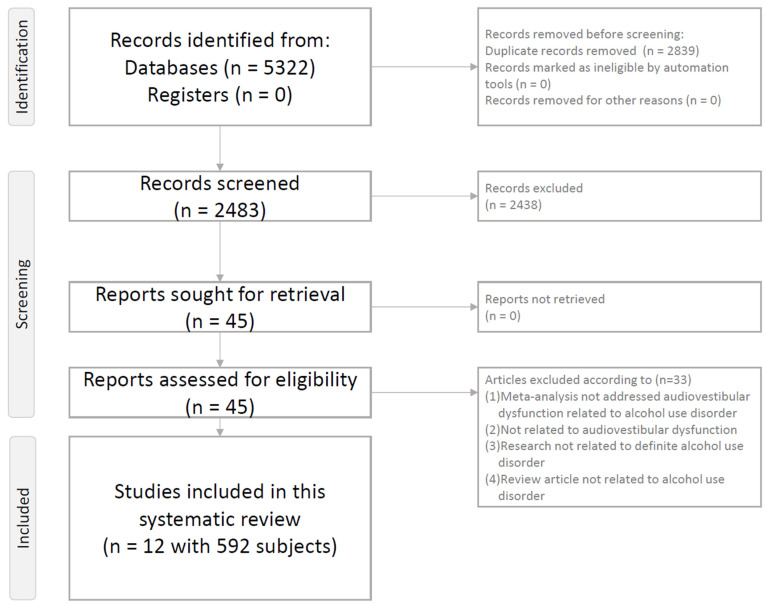
PRISMA 2020 flow diagram of the study selection process.

After removal of duplicates and ineligible records [2,4,8,9,10,11,14,16,18,19,20,21,22,23,24,25,26,27,28,29,30,31,32,33,34,35,36,37,38,39,40,41,42], 12 human primary clinical studies were included in the final qualitative synthesis (Figure 1). The PRISMA flow diagram is shown in Figure 1, and study-level characteristics are summarized in Tables S4 and S5.

Figure 1 illustrates the study identification, screening, eligibility assessment, and final inclusion process for the present systematic review.

### 2.1. Study Characteristics and Methodological Quality

The included evidence base was dominated by small human primary clinical studies, most of which were single-center observational investigations or descriptive case reports/series [1,5,6,15,43,44,45,46,47,48,49,50]. No randomized controlled trial and no sufficiently homogeneous dataset for quantitative synthesis were identified. Sample sizes ranged from single-patient case reports to comparative studies including up to 118 participants [15,50], and only a minority of studies included a control group. Alcohol-related clinical contexts also varied across studies, including chronic alcoholism, alcohol dependence, alcohol abuse, abstinent alcoholism, and alcohol-related Wernicke/Wernicke–Korsakoff presentations [1,5,15,43,45,46,49,50].

Outcome ascertainment was heterogeneous and included self-reported symptoms, pure-tone audiometry, otoacoustic emissions, acoustic reflex testing, central auditory processing tests, auditory brainstem responses, and nystagmographic/electronystagmographic assessments [1,6,44,45,46,48,49,50]. Overall methodological quality was low to moderate. The main limitations were small sample size, limited control for confounding, inconsistent diagnostic definitions, incomplete specification of alcohol exposure severity and duration, and reliance on historical audiovestibular test batteries.

### 2.2. Auditory Manifestations

Across the included human clinical literature, the most consistent auditory finding was sensorineural hearing dysfunction, often with greater involvement at higher frequencies [1,5,6,43,44,46]. Prevalence estimates varied widely across studies because of differences in study populations, diagnostic thresholds, abstinence status, and testing methods, but objective hearing abnormalities were generally reported more frequently than subjective complaints [1,5,6,46]. In clinical cohorts of chronic alcohol dependence or alcoholism, hearing impairment was documented on pure-tone audiometry in a substantial proportion of participants, whereas self-reported symptoms underestimated the burden of dysfunction [1,46].

The pattern of abnormalities suggested that auditory dysfunction in alcohol use disorder may extend beyond simple cochlear threshold shifts. Studies using otoacoustic emissions, acoustic reflex testing, and central auditory processing measures reported findings compatible with both cochlear and retrocochlear involvement [6,44,46,48]. Auditory brainstem response studies likewise identified prolonged inter-wave intervals and other electrophysiologic abnormalities, particularly among patients with long-standing alcohol-related neurologic involvement [49,50]. Taken together, the direction of evidence consistently supported auditory dysfunction in alcohol use disorder, although heterogeneity in outcome definitions and study design precluded quantitative synthesis.

### 2.3. Vestibular Manifestations

The vestibular evidence base was smaller and phenotypically less specific than the auditory literature. In the principal longitudinal clinical cohort focused on vestibular outcomes, peripheral, central, and combined vestibular disorders were all reported, with combined abnormalities being more common in older individuals and in those with longer drinking histories [45]. In another clinical series, vertigo and related audiovestibular complaints were reported in a subset of patients with alcohol dependence, but objective findings suggested a greater burden of dysfunction than symptom reporting alone [1].

Importantly, most included studies did not classify patients according to contemporary vestibular diagnostic entities. Instead, the literature primarily described symptoms (e.g., vertigo, imbalance), nystagmographic abnormalities, or broad peripheral/central patterns [1,45]. Therefore, while the available clinical evidence supports vestibular involvement in alcohol use disorder, it does not permit robust phenotype-level classification of specific vestibular disorders.

### 2.4. Diagnostic Findings and Limited Pathophysiologic Correlates

Within the included human clinical literature, diagnostic evidence relied predominantly on conventional audiometry and legacy neuro-otologic test batteries. The most frequently reported auditory assessments were pure-tone audiometry, impedance/acoustic reflex testing, otoacoustic emissions, and auditory brainstem responses [1,6,44,46,48,49,50]. Across studies, objective testing often identified dysfunction even when patients did not clearly report hearing symptoms [1,46]. These data support the view that subclinical or under-recognized auditory impairment may be present in alcohol use disorder.

Vestibular assessment in the included literature was more limited and largely based on nystagmographic or electronystagmographic methods [1,45]. The reported abnormalities suggested that both peripheral and central vestibular pathways may be involved, but the evidence remained insufficiently detailed for precise clinical phenotyping. Direct pathophysiologic evidence from human primary studies was also sparse. Available clinicopathologic and neurophysiologic reports suggested possible cochlear, eighth-nerve, brainstem, and combined pathway involvement [47,48,49,50], but no included clinical study could reliably disentangle the relative contributions of direct ethanol toxicity, nutritional deficiency, and broader alcohol-related neurologic comorbidity.

### 2.5. Treatment and Prognosis

Direct treatment evidence was very limited. No controlled human study evaluated a disease-specific treatment for audiovestibular dysfunction attributable to alcohol use disorder. Therapeutic observations were confined mainly to isolated case-based evidence and syndromic alcohol-related presentations. In alcohol-related Wernicke encephalopathy with hearing involvement, improvement following thiamine replacement has been reported [15]. However, the currently available human clinical literature does not support any established disease-specific pharmacologic or procedural treatment for alcohol use disorder-related audiovestibular dysfunction.

Prognostic data were similarly sparse. In longitudinal follow-up, vestibular abnormalities showed partial reversibility after sustained abstinence [45], whereas auditory abnormalities in some abstinent individuals persisted at selected frequencies despite alcohol cessation [46]. Overall, the human clinical evidence suggests that some deficits may improve when reversible contributors are addressed, but persistent residual dysfunction can occur, particularly in long-standing disease.

## 3. Discussion

In the present review, the human primary clinical evidence supported the presence of both auditory and vestibular abnormalities in alcohol use disorder; however, the evidence base was small, methodologically heterogeneous, and often insufficiently specific for phenotype-level vestibular classification or disease-specific treatment recommendations. Across the included studies, the most consistent signal involved objective auditory dysfunction, particularly sensorineural hearing impairment and high-frequency abnormalities, whereas vestibular involvement was supported by a smaller and less specific literature composed mainly of syndromic reports, broad peripheral/central classifications, and historical nystagmographic findings [1,5,6,15,43,44,45,46,47,48,49,50].

**Figure 2:**
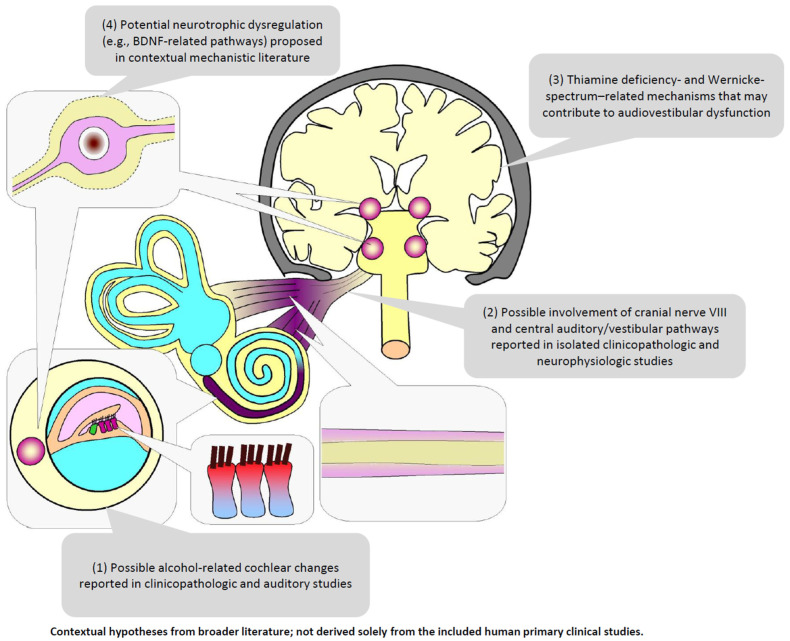
Contextual mechanistic hypotheses potentially contributing to audiovestibular dysfunction in alcohol use disorder.

An important implication of the present synthesis is that the currently available human clinical literature does not allow firm separation of the relative contributions of direct ethanol-related ototoxicity, nutritional deficiency, Wernicke/Wernicke–Korsakoff-related injury, and broader alcohol-associated neurologic comorbidity. Some included studies suggested cochlear, eighth-nerve, brainstem, or combined pathway involvement [47,48,49,50], but these observations arose from small and methodologically diverse clinical datasets. Therefore, although contextual clinicopathologic and mechanistic literature supports biologically plausible pathways involving thiamine deficiency, neurotoxicity, neuroinflammation, and neurotrophic dysregulation [14,29,35,36,37,38,39,51,52,53,54,55,56,57,58], these mechanisms should be interpreted as explanatory background rather than as conclusions directly established by the included human primary clinical studies. We summarized the conceptual framework of possible mechanisms underlying audiovestibular dysfunction in alcohol use disorder in Figure 2. A contextual overview of these proposed mechanistic hypotheses is shown in Figure 2.

Figure 2 summarizes broader contextual mechanisms discussed in the manuscript and should not be interpreted as conclusions derived solely from the included human primary clinical studies. Panel (1) reflects clinicopathologic and auditory evidence suggesting possible cochlear involvement [29,46]. Panel (2) reflects isolated human clinicopathologic and neurophysiologic reports suggesting possible involvement of cranial nerve VIII and central auditory/vestibular pathways [47,48,49,50]. Panel (3) summarizes thiamine deficiency- and Wernicke-spectrum mechanisms discussed in the background literature [14,15,17]. Panel (4) reflects contextual literature suggesting potential neurotrophic dysregulation, including BDNF-related pathways [51,52,53]. Relevant supporting references for each mechanistic domain are provided in the main text where Figure 2 is first introduced.

Beyond direct physiologic mechanisms, behavioral and environmental cofactors may also contribute to the observed burden of audiovestibular dysfunction in alcohol use disorder. Individuals with harmful alcohol use may be repeatedly exposed to loud leisure-noise environments such as bars, nightclubs, concerts, festivals, and sporting venues, which are themselves recognized sources of hearing injury [59]. In music-event settings, simultaneous alcohol/drug use and nonuse of earplugs have been associated with temporary noise-induced hearing loss among attendees [60]. Accordingly, part of the audiological burden observed in alcohol use disorder may reflect combined effects of ethanol exposure and recurrent leisure-noise co-exposure rather than a purely alcohol-specific biologic mechanism.

The diagnostic literature identified in this review was also shaped by the eras in which many of the included studies were conducted. As a result, the historical evidence base relied heavily on pure-tone audiometry, acoustic reflex testing, auditory brainstem responses, electronystagmography, and older retrocochlear-oriented batteries such as SISI, tone decay, and fixed-frequency Békésy testing [1,6,44,45,48,49,50]. These methods remain informative for understanding how earlier investigators approached alcohol-related audiovestibular dysfunction, but they should not be interpreted as routine contemporary standards of care. Modern vestibular practice emphasizes a syndrome-oriented and lesion-oriented approach in which test selection is guided by the clinical phenotype and the suspected anatomic substrate, rather than by indiscriminate use of a fixed battery [61,62,63,64,65,66,67]. In this framework, vHIT, VEMP, caloric testing, and modern nystagmus-based assessment provide complementary rather than interchangeable information, and abnormal findings may dissociate according to the frequency domain tested and the vestibular end organ involved [63,64,65,66,67,68]. Accordingly, the older vestibular tools reported in the historical alcohol literature are best viewed as part of the descriptive evidence base, whereas current clinical evaluation should preferentially use symptom-guided and phenotype-guided audiovestibular assessment.

The treatment literature was particularly limited. No controlled human clinical study evaluated a disease-specific treatment for audiovestibular dysfunction attributable to alcohol use disorder. Improvement after thiamine replacement has been reported in alcohol-related Wernicke-spectrum presentations with hearing or vestibular involvement [15], which is clinically plausible given the established role of thiamine deficiency in alcohol-related neurologic injury [12,13,14]. However, beyond such syndromic situations, the present review did not identify robust human evidence supporting any specific pharmacologic or procedural therapy for alcohol use disorder-related audiovestibular dysfunction. The previous manuscript version may have overstated the translational relevance of magnesium or neurotrophic approaches. In the revised interpretation, these strategies should be regarded as mechanistic or experimental considerations rather than clinically supported treatments [24,25,33,35,36,37,38,39,54,55,56,57,58,69,70,71].

Taken together, the present review supports a cautious clinical interpretation. Alcohol use disorder appears to be associated with clinically relevant auditory dysfunction and probable vestibular involvement, but the evidence remains too heterogeneous and historically fragmented to justify disease-specific screening algorithms, legacy test batteries as routine recommendations, or claims regarding established targeted therapies. The most defensible conclusion is therefore one of heightened clinical awareness rather than therapeutic certainty.

### 3.1. Strengths and Limitations

This review has several strengths. First, the synthesis was restricted to human primary clinical evidence, which improved conceptual consistency between the eligibility criteria, the synthesis methods, and the conclusions. Second, the Results were reorganized into explicit synthesis domains rather than a sequential narrative of individual studies. Third, study design, sample size limitations, and methodological constraints are now integrated into the main Results section, allowing a more transparent interpretation of the evidence base.

Several limitations remain important. The available literature was sparse and dominated by small observational studies and descriptive case reports/series, many of which were conducted decades ago using historical diagnostic methods [1,5,6,15,43,44,45,46,47,48,49,50]. Definitions of alcohol-related exposure varied across studies, including alcoholism, alcohol dependence, alcohol abuse, abstinent alcoholism, and Wernicke/Wernicke–Korsakoff-related presentations, which limited comparability. The vestibular literature was particularly underdeveloped, with few studies providing contemporary phenotype-based classification of specific vestibular disorders. Potential confounding by smoking, occupational or leisure-noise exposure, polysubstance use, malnutrition, prior falls or head trauma, liver disease, cerebral atrophy, and other neurologic comorbidities was often insufficiently addressed [59,60]. Because outcomes, populations, and methods were highly heterogeneous, quantitative meta-analysis was not appropriate. Finally, some mechanistic discussions necessarily relied on contextual non-included literature, which can support biological plausibility but cannot substitute for direct clinical evidence.

### 3.2. Clinical Implications

The present review supports a pragmatic and restrained clinical message. Clinicians caring for patients with alcohol use disorder should maintain awareness of possible hearing loss, tinnitus, vertigo, imbalance, and oscillopsia, particularly when such symptoms occur in the setting of malnutrition, gait disturbance, nystagmus, or other neurologic features suggestive of alcohol-related brain involvement. When audiovestibular symptoms are present, evaluation should be individualized and guided by the dominant clinical phenotype, with targeted use of contemporary audiologic and vestibular testing when clinically indicated rather than routine use of legacy test batteries [61,62,63,64,65,66,67,68]. However, the current evidence does not justify a disease-specific screening protocol or the routine use of historical audiovestibular batteries derived from older literature.

Instead, in patients with suspected Wernicke encephalopathy or Wernicke–Korsakoff syndrome, prompt recognition and thiamine replacement remain clinically important because some neurologic and audiovestibular abnormalities may be at least partially reversible [12,13,14,15]. By contrast, the present evidence does not support routine recommendation of experimental neurotrophic therapies or disease-specific pharmacologic strategies for alcohol use disorder-related audiovestibular dysfunction.

### 3.3. Future Directions

Future studies should move beyond broad descriptive labels and adopt standardized alcohol-related diagnostic definitions, clearer phenotyping of auditory and vestibular outcomes, and more explicit control of major confounders. In particular, prospective cohort studies using contemporary audiologic and vestibular assessment frameworks would help determine whether alcohol use disorder is associated with reproducible phenotype-level vestibular patterns, whether certain deficits are reversible with abstinence or nutritional correction, and which abnormalities reflect direct ethanol toxicity versus secondary neurologic or nutritional injury. Studies that separately evaluate current alcohol exposure, abstinence duration, Wernicke-related features, and polysubstance use would be especially valuable.

Another underexplored pathway is indirect injury mediated by alcohol-related imbalance, falls, and head or neck trauma. Experimental human studies have shown that alcohol intoxication impairs postural control [72], alcohol-related falls are associated with more severe craniofacial and head injury [73], and vestibular dysfunction is common after traumatic brain injury [74]. Future alcohol use disorder-focused studies should therefore record prior falls, head/neck trauma, and noisy leisure-environment exposures, and should examine whether some audiovestibular abnormalities attributed to alcohol use disorder are mediated or amplified by recurrent trauma and environmental co-exposures.

## 4. Methods and Materials

### 4.1. Study Design and Registration

This study was conducted as a systematic review of human primary clinical evidence on audiovestibular dysfunction in alcohol use disorder. The review followed the Preferred Reporting Items for Systematic Reviews and Meta-Analyses (PRISMA) statement [75] and was prospectively registered in PROSPERO (CRD420261301021). The PRISMA checklist is provided (Table S1), and study selection process is shown in Figure 1.

To maintain conceptual consistency between the review objective, eligibility criteria, synthesis strategy, and conclusions, only human primary clinical studies were included in the final synthesis. Non-human, in vitro, review-level, and other non-primary evidence sources were excluded.

### 4.2. Search Strategy

A comprehensive search of PubMed, Embase, ClinicalKey, Web of Science, and ScienceDirect was performed from inception to 4 February 2026. The search strategy combined controlled vocabulary and free-text terms related to alcohol use disorder and audiovestibular dysfunction, with database-specific adaptations as appropriate. The full search strategy is shown in Table S2.

To ensure comprehensive identification of relevant human primary clinical evidence, we also manually reviewed the reference lists of included articles. No language or publication date restrictions were applied at the search stage. Excluded full-text articles and the reasons for exclusion are listed in Table S3.

### 4.3. Eligibility Criteria

We included human primary clinical studies reporting auditory, vestibular, or combined audiovestibular dysfunction in patients with alcohol use disorder or historically related clinical diagnoses, including alcohol dependence, alcohol abuse, or alcoholism. Eligible study designs included case reports, case series, cross-sectional studies, case-control studies, cohort studies, and randomized controlled trials. Studies were required to report at least one clinically relevant domain, including manifestations, diagnostic findings, treatment, or prognosis of audiovestibular dysfunction.

We excluded animal studies, cell-line or other in vitro studies, review articles, systematic reviews, meta-analyses, narrative reviews, editorials, and studies without primary human clinical data. We also excluded studies focused on alcohol consumption in the general population without a clinically relevant alcohol use disorder population, as well as studies not reporting relevant auditory or vestibular outcomes.

Given historical variation in diagnostic terminology, older studies using terms such as alcoholism, alcohol dependence, or alcohol abuse were considered eligible when the study population clearly represented a clinically significant alcohol-related disorder. Alcohol-related Wernicke or Wernicke–Korsakoff studies were included only if they provided primary human clinical evidence directly relevant to audiovestibular dysfunction. Excluded articles are listed in Table S3.

### 4.4. Screening and Selection

Two authors (PT Tseng, YW Chen) independently screened all retrieved titles and abstracts, followed by full-text assessment of potentially eligible records according to the predefined inclusion and exclusion criteria. Duplicate records were removed using reference management software and manually checked for accuracy. Any disagreement during screening or full-text review was resolved through discussion and, when needed, consultation with a third author (JJ Chen). The study selection process is summarized in Figure 1.

### 4.5. Data Extraction

Two authors (PT Tseng and YW Chen) independently extracted data from each included study using a standardized approach. Extracted variables included study characteristics, patient population, alcohol-related clinical context, auditory and vestibular findings, diagnostic methods, treatment information, prognostic observations, and major methodological features relevant to synthesis. Where available, additional details regarding comparator groups, abstinence status, and Wernicke- or Wernicke–Korsakoff-related features were also collected. Discrepancies in data extraction were resolved by discussion and consensus.

### 4.6. Quality Assessment

Study quality was independently assessed by PT Tseng and YW Chen using design-appropriate critical appraisal tools. Cohort and case-control studies were evaluated using the Newcastle-Ottawa Scale [76,77], randomized controlled trials, if identified, using the Cochrane Risk of Bias tool, and case reports/case series using design-appropriate critical appraisal checklists. Disagreements were resolved through discussion and, when required, consultation with a third author. Quality assessment findings were used to inform interpretation of the evidence but were not used as exclusion criteria. The results of critical appraisal are summarized in Table S4.

### 4.7. Data Synthesis

Because the included studies showed substantial heterogeneity in design, alcohol-related clinical context, outcome definitions, and audiovestibular assessment methods, meta-analysis was not appropriate. We therefore performed a structured qualitative synthesis.

Studies were grouped a priori into the following synthesis domains: study characteristics and methodological quality, auditory manifestations, vestibular manifestations, diagnostic findings and limited pathophysiologic correlates, and treatment and prognosis. Within these domains, studies were further organized by design and clinical context to facilitate interpretation.

For each synthesis domain, we summarized study design, sample size, patient characteristics, diagnostic methods, principal findings, consistency in direction of results, and major methodological limitations. Given the predominance of small and heterogeneous descriptive studies, the synthesis focused on recurrent clinical patterns, areas of convergence, and evidence gaps rather than quantitative effect estimation. Critical appraisal findings informed interpretation of the strengths and limitations of the evidence base.

## 5. Conclusions

Available human primary clinical evidence suggests that alcohol use disorder is associated with auditory dysfunction and probable vestibular involvement. However, the evidence base is small, heterogeneous, and often based on older descriptive studies using non-uniform methods. Current data support heightened clinical awareness and context-sensitive audiovestibular evaluation when symptoms are present, but they do not support disease-specific screening algorithms, routine use of legacy vestibular test batteries, or established targeted therapies. Better-designed prospective human studies using contemporary phenotype-based audiovestibular assessment are needed to clarify mechanisms, refine diagnosis, and guide management.

## Data Availability

No new data were created or analyzed in this study. Data sharing is not applicable to this article.

## Supplementary Materials

The following supporting information can be downloaded at: https://www.mdpi.com/article/10.3390/ijms27093905/s1, References [1,2,4,5,6,8,9,10,11,14,15,16,18,19,20,21,22,23,24,25,26,27,28,29,30,31,32,33,34,35,36,37,38,39,40,41,42,43,44,45,46,47,48,49,50,75] are cited in the Supplementary Material.
