# Supplementary material for: Audiovestibular Dysfunction in Alcohol Use Disorder: A Systematic Review of Human Primary Clinical Evidence
Source: Int J Mol Sci. 2026 Apr 28;27(9):3905. doi: 10.3390/ijms27093905 (PMC13164321; doi:10.3390/ijms27093905)
Supplement: Supplementary file 1 [file ijms-27-03905-s001.zip › ijms-4231553-supplementary.pdf]

Table S1: PRISMA 2020 checklist of current systematic review

Section and Topic	Item #	Checklist item	Page where item is reported
**TITLE**			
Title	1	Identify the report as a systematic review.	1
**ABSTRACT**			
Abstract	2	See the PRISMA 2020 for Abstracts checklist.	1-2
**INTRODUCTION**			
Rationale	3	Describe the rationale for the review in the context of existing knowledge.	2
Objectives	4	Provide an explicit statement of the objective(s) or question(s) the review addresses.	2
**METHODS**			
Eligibility criteria	5	Specify the inclusion and exclusion criteria for the review and how studies were grouped for the syntheses.	9
Information sources	6	Specify all databases, registers, websites, organisations, reference lists and other sources searched or consulted to identify studies. Specify the date when each source was last searched or consulted.	9
Search strategy	7	Present the full search strategies for all databases, registers and websites, including any filters and limits used.	9
Selection process	8	Specify the methods used to decide whether a study met the inclusion criteria of the review, including how many reviewers screened each record and each report retrieved, whether they worked independently, and if applicable, details of automation tools used in the process.	9
Data collection process	9	Specify the methods used to collect data from reports, including how many reviewers collected data from each report, whether they worked independently, any processes for obtaining or confirming data from study investigators, and if applicable, details of automation tools used in the process.	9
Data items	10a	List and define all outcomes for which data were sought. Specify whether all results that were compatible with each outcome domain in each study were sought (e.g. for all measures, time points, analyses), and if not, the methods used to decide which results to collect.	10
	10b	List and define all other variables for which data were sought (e.g. participant and intervention characteristics, funding sources). Describe any assumptions made about any missing or unclear information.	10
Study risk of bias assessment	11	Specify the methods used to assess risk of bias in the included studies, including details of the tool(s) used, how many reviewers assessed each study and whether they worked independently, and if applicable, details of automation tools used in the process.	10
Effect measures	12	Specify for each outcome the effect measure(s) (e.g. risk ratio, mean difference) used in the synthesis or presentation of results.	10
Synthesis methods	13a	Describe the processes used to decide which studies were eligible for each synthesis (e.g. tabulating the study intervention characteristics and comparing against the planned groups for each synthesis (item #5)).	10
	13b	Describe any methods required to prepare the data for presentation or synthesis, such as handling of missing summary statistics, or data conversions.	10

Section and Topic	Item #	Checklist item	Page where item is reported
	13c	Describe any methods used to tabulate or visually display results of individual studies and syntheses.	10
	13d	Describe any methods used to synthesize results and provide a rationale for the choice(s). If meta-analysis was performed, describe the model(s), method(s) to identify the presence and extent of statistical heterogeneity, and software package(s) used.	10
	13e	Describe any methods used to explore possible causes of heterogeneity among study results (e.g. subgroup analysis, meta-regression).	10
	13f	Describe any sensitivity analyses conducted to assess robustness of the synthesized results.	10
Reporting bias assessment	14	Describe any methods used to assess risk of bias due to missing results in a synthesis (arising from reporting biases).	10
Certainty assessment	15	Describe any methods used to assess certainty (or confidence) in the body of evidence for an outcome.	10
**RESULTS**			
Study selection	16a	Describe the results of the search and selection process, from the number of records identified in the search to the number of studies included in the review, ideally using a flow diagram.	2-3, Figure 1
	16b	Cite studies that might appear to meet the inclusion criteria, but which were excluded, and explain why they were excluded.	2-3
Study characteristics	17	Cite each included study and present its characteristics.	2-3, Table S5
Risk of bias in studies	18	Present assessments of risk of bias for each included study.	Table S4
Results of individual studies	19	For all outcomes, present, for each study: (a) summary statistics for each group (where appropriate) and (b) an effect estimate and its precision (e.g. confidence/credible interval), ideally using structured tables or plots.	3, Table S5
Results of syntheses	20a	For each synthesis, briefly summarise the characteristics and risk of bias among contributing studies.	4-5
	20b	Present results of all statistical syntheses conducted. If meta-analysis was done, present for each the summary estimate and its precision (e.g. confidence/credible interval) and measures of statistical heterogeneity. If comparing groups, describe the direction of the effect.	4-5, Table S5
	20c	Present results of all investigations of possible causes of heterogeneity among study results.	Table S5
	20d	Present results of all sensitivity analyses conducted to assess the robustness of the synthesized results.	4-5
Reporting biases	21	Present assessments of risk of bias due to missing results (arising from reporting biases) for each synthesis assessed.	4-5
Certainty of evidence	22	Present assessments of certainty (or confidence) in the body of evidence for each outcome assessed.	4-5
**DISCUSSION**			
Discussion	23a	Provide a general interpretation of the results in the context of other evidence.	5-6
	23b	Discuss any limitations of the evidence included in the review.	6-7
	23c	Discuss any limitations of the review processes used.	6-7

Section and Topic	Item #	Checklist item	Page where item is reported
	23d	Discuss implications of the results for practice, policy, and future research.	7
**OTHER INFORMATION**			
Registration and protocol	24a	Provide registration information for the review, including register name and registration number, or state that the review was not registered.	9
	24b	Indicate where the review protocol can be accessed, or state that a protocol was not prepared.	9
	24c	Describe and explain any amendments to information provided at registration or in the protocol.	9
Support	25	Describe sources of financial or non-financial support for the review, and the role of the funders or sponsors in the review.	10-11
Competing interests	26	Declare any competing interests of review authors.	10-11
Availability of data, code and other materials	27	Report which of the following are publicly available and where they can be found: template data collection forms; data extracted from included studies; data used for all analyses; analytic code; any other materials used in the review.	10-11

The current checklist followed the latest PRISMA 2020 guideline [75].

Table S2: Keyword and search results in each database

Database	Keyword	Filter	Date	Result
PubMed	("alcohol use disorder" OR "Alcohol Abuse" OR "Alcohol Dependence") AND (hearing loss OR sensorineural hearing loss OR SNHL OR audiology OR tinnitus OR vertigo OR vestibular OR dizziness)	N/A	2026/02/04	142
Embase	("alcohol use disorder" OR "Alcohol Abuse" OR "Alcohol Dependence") AND (hearing loss OR sensorineural hearing loss OR SNHL OR audiology OR tinnitus OR vertigo OR vestibular OR dizziness)	N/A	2026/02/04	2347
ClinicalKey	("alcohol use disorder" OR "Alcohol Abuse" OR "Alcohol Dependence") AND (hearing loss OR sensorineural hearing loss OR SNHL OR audiology OR tinnitus OR vertigo OR vestibular OR dizziness)	N/A	2026/02/04	327
Web of Science	("alcohol use disorder" OR "Alcohol Abuse" OR "Alcohol Dependence") AND (hearing loss OR sensorineural hearing loss OR SNHL OR audiology OR tinnitus OR vertigo OR vestibular OR dizziness)	N/A	2026/02/04	100
ScienceDirect	(“alcohol use disorder”) AND (hearing loss OR sensorineural hearing loss OR SNHL OR audiology OR tinnitus OR vertigo OR vestibular OR dizziness)	N/A	2026/02/04	2406

Abbreviation: N/A: not applied

Table S3: Excluded studies and reason

Reason	Numbers	References
Meta-analysis not addressed audiovestibular dysfunction related to alcohol use disorder	2	[2,23]
Not related to audiovestibular dysfunction	10	[14,16,20,21,24-29]
Research not related to definite alcohol use disorder	17	[4,8-11,18,19,30-39]
Review article not related to alcohol use disorder	10	[22,40-42]

Table S4: Newcastle-Ottawa Scale and Characteristics for the Included Trial (observational trial)

Study	**Selection** Case definition	Representative	Control selection	Control definition	**Comparability** Comparability	**Exposure** Ascertainment	Same method	Non-Response rate	Total Summary
Chan, Y.W. (1985)[50]	*	*	*	*	*	*	*		7*
Chu, N.S. (1982)[49]	*	*				*		*	4*
Golabek, W. (1984)[44]	*	*				*		*	4*
Morgan, Z.J. (2020)[15]	*	*				*			3*
Niedzielska, G. (2001)[6]	*	*				*		*	4*
Nordahl, T. (1964)[5]	*	*				*		*	4*
Ribeiro, S.B. (2007)[46]	*	*				*		*	4*
Sasa, M. (1981)[45]	*	*	*	*	*	*	*		7*
Spitzer, J.B. (1980)[48]	*	*	*	*	*	*	*		7*
Verma, R.K. (2006)[1]	*	*				*		*	4*
Wheeler, D.C. (1980)[43]	*	*	*	*		*		*	6*
Ylikoski, J.S. (1981)[47]	*	*				*			3*

* indicated this study have a good performance in this item

Table S5: Summary of the included study

Study name	Study design	Population	Key audiovestibular assessments	Main findings	Key limitations
Chan, Y.W. (1985)[50]	Case-control	25 alcoholics with Wernicke–Korsakoff syndrome, 56 alcoholics without the syndrome, and 37 controls	BAER	BAER abnormalities were more frequent in the Wernicke–Korsakoff group; prolonged I–III and I–V intervals were more common	Findings were strongly influenced by Wernicke–Korsakoff status
Chu, N.S. (1982)[49]	Cross-sectional	66 chronic alcoholic patients	ABR; CT correlation in some patients	Prolonged interwave intervals were reported in 41%; abnormalities were associated with neurologic complications and cerebral atrophy	No non-alcoholic comparator; CNS comorbidity likely relevant
Golabek, W. (1984)[44]	Cross-sectional	67 individuals with chronic alcoholism	PTA; impedance audiometry; SISI; tone decay; fixed-frequency Békésy in a subset	Sensorineural hearing loss was reported; findings in a subset were interpreted as suggestive of retrocochlear involvement	Historical test battery; no control group; indirect lesion inference
Morgan, Z.J. (2020)[15]	Case report	46-year-old woman with alcohol abuse–related Wernicke encephalopathy and bilateral deafness	Clinical neurologic and hearing evaluation	Bilateral hearing loss improved after thiamine replacement	Single case; Wernicke-spectrum presentation
Niedzielska, G. (2001)[6]	Cross-sectional	30 individuals with chronic alcoholism	PTA; impedance audiometry; OAE; ABR	Sensorineural hearing loss, absent otoacoustic emissions, and brainstem auditory abnormalities were reported	Small sample; no control group
Nordahl, T. (1964)[5]	Cross-sectional	83 alcoholics after exclusion of chronic otitis media and major streptomycin exposure	Hearing examination; audiometric survey	Hearing loss was identified in a subset of participants; alcohol was discussed as a possible contributing factor	Early historical study; confounding insufficiently controlled
Ribeiro, S.B. (2007)[46]	Case-control	75 individuals in alcoholic and control groups	Audiologic assessment, including PTA and OAE-based cochlear evaluation	Worse audiologic performance was observed in the alcoholic group; findings were compatible with cochlear dysfunction and outer hair-cell involvement	Modest sample; limited control of confounders
Sasa, M. (1981)[45]	Cohort with follow-up	33 Japanese men with long-term alcoholism; 16 controls;	Vestibular assessment with peripheral/central/combined classification	Peripheral, central, and combined vestibular abnormalities were reported; partial	Small cohort; outdated vestibular classification

		follow-up subset after abstinence		improvement was observed after prolonged abstinence	
Spitzer, J.B. (1980)[48]	Case-control	15 alcoholic subjects and 15 age-matched controls	Acoustic reflexes; SSW; SSI; temporal summation; standard audiologic tests	Abnormal central auditory findings were reported; results were interpreted as compatible with brainstem dysfunction in some subjects	Small sample; historical central auditory battery
Verma, R.K. (2006)[1]	Case-control	20 patients with long-term alcohol dependence	PTA; BERA; ENG in symptomatic cases	High-frequency threshold elevation, abnormal brainstem auditory findings, and abnormal ENG findings in some participants with vertigo were reported	Small sample; limited vestibular specificity; historical methods
Wheeler, D.C. (1980)[43]	Cross-sectional	52 alcoholic subjects	Audiometric configuration analysis	Bilateral high-frequency hearing loss was associated with drinking duration	No control group; possible residual confounding
Ylikoski, J.S. (1981)[47]	Case report	55-year-old man with hearing loss, balance disturbance, facial weakness, chronic alcoholism, and nutritional deficiency	Clinical evaluation; histopathology of cranial nerve VIII	Degeneration of cochlear and vestibular nerve fibers was reported	Single confounded case; limited generalizability

Reference list of supplement tables:

1. Verma, R.K.; Panda, N.K.; Basu, D.; Raghunathan, M. Audiovestibular dysfunction in alcohol dependence. Are we worried? *Am J Otolaryngol* **2006**, *27*, 225-228, doi:10.1016/j.amjoto.2005.09.005.
2. Qian, P.; Zhao, Z.; Liu, S.; Xin, J.; Liu, Y.; Hao, Y.; Wang, Y.; Yang, L. Alcohol as a risk factor for hearing loss: A systematic review and meta-analysis. *PloS one* **2023**, *18*, e0280641, doi:10.1371/journal.pone.0280641.
4. Nakamura, M.; Aoki, N.; Nakashima, T.; Hoshino, T.; Yokoyama, T.; Morioka, S.; Kawamura, T.; Tanaka, H.; Hashimoto, T.; Ohno, Y.; et al. Smoking, alcohol, sleep and risk of idiopathic sudden deafness: a case-control study using pooled controls. *Journal of epidemiology* **2001**, *11*, 81-86, doi:10.2188/jea.11.81.
5. Nordahl, T. Examination of Hearing in Alcoholics. *Acta Otolaryngol Suppl* **1964**, *188*, SUPPL 188:362+, doi:10.3109/00016486409134589.
6. Niedzielska, G.; Katska, E.; Kusa, W. Hearing loss in chronic alcoholics. *Ann Univ Mariae Curie Sklodowska Med* **2001**, *56*, 99-101.
8. Curhan, S.G.; Eavey, R.; Wang, M.; Stampfer, M.J.; Curhan, G.C. Prospective study of alcohol consumption and self-reported hearing loss in women. *Alcohol* **2015**, *49*, 71-77, doi:10.1016/j.alcohol.2014.10.001.
9. Upile, T.; Sipaul, F.; Jerjes, W.; Singh, S.; Nouraei, S.A.; El Maaytah, M.; Andrews, P.; Graham, J.; Hopper, C.; Wright, A. The acute effects of alcohol on auditory thresholds. *BMC Ear Nose Throat Disord* **2007**, *7*, 4, doi:10.1186/1472-6815-7-4.
10. Hwang, J.H.; Tan, C.T.; Chiang, C.W.; Liu, T.C. Acute effects of alcohol on auditory thresholds and distortion product otoacoustic emissions in humans. *Acta Otolaryngol* **2003**, *123*, 936-940, doi:10.1080/00016480310014877.
11. Jozefowicz-Korczynska, M.; Lukomski, M.; Kurnatowski, P. Vestibular system in alcohol and drug addicts. *Mater Med Pol* **1994**, *26*, 123-126.
14. Martin, P.R.; Singleton, C.K.; Hiller-Sturmhofel, S. The role of thiamine deficiency in alcoholic brain disease. *Alcohol Res Health* **2003**, *27*, 134-142.
15. Morgan, Z.J.; Cler, L.; Hunter, L. I can't hear you, you said I had what?: A case report and literature review. *J Community Hosp Intern Med Perspect* **2020**, *10*, 443-445, doi:10.1080/20009666.2020.1808359.

16. Todd, K.G.; Butterworth, R.F. Evaluation of the role of NMDA-mediated excitotoxicity in the selective neuronal loss in experimental Wernicke encephalopathy. *Exp Neurol* **1998**, *149*, 130-138, doi:10.1006/exnr.1997.6677.
18. Popelka, M.M.; Cruickshanks, K.J.; Wiley, T.L.; Tweed, T.S.; Klein, B.E.; Klein, R.; Nondahl, D.M. Moderate alcohol consumption and hearing loss: a protective effect. *J Am Geriatr Soc* **2000**, *48*, 1273-1278, doi:10.1111/j.1532-5415.2000.tb02601.x.
19. Dawes, P.; Cruickshanks, K.J.; Moore, D.R.; Edmondson-Jones, M.; McCormack, A.; Fortnum, H.; Munro, K.J. Cigarette smoking, passive smoking, alcohol consumption, and hearing loss. *J Assoc Res Otolaryngol* **2014**, *15*, 663-674, doi:10.1007/s10162-014-0461-0.
20. Miller, A.P.; Kuo, S.I.; Johnson, E.C.; Tillman, R.; Brislin, S.J.; Dick, D.M.; Kamarajan, C.; Kinreich, S.; Kramer, J.; McCutcheon, V.V.; et al. Diagnostic Criteria for Identifying Individuals at High Risk of Progression From Mild or Moderate to Severe Alcohol Use Disorder. *JAMA Netw Open* **2023**, *6*, e2337192, doi:10.1001/jamanetworkopen.2023.37192.
21. Rehm, J.; Baliunas, D.; Borges, G.L.; Graham, K.; Irving, H.; Kehoe, T.; Parry, C.D.; Patra, J.; Popova, S.; Poznyak, V.; et al. The relation between different dimensions of alcohol consumption and burden of disease: an overview. *Addiction* **2010**, *105*, 817-843, doi:10.1111/j.1360-0443.2010.02899.x.
22. Chiao, A.; Hughes, M.L.; Karimuddanahalli Premkumar, P.; Zoucha, K. The Effects of Substance Misuse on Auditory and Vestibular Function: A Systematic Review. *Ear Hear* **2024**, *45*, 276-296, doi:10.1097/AUD.0000000000001425.
23. Kranzler, H.R.; Feinn, R.; Morris, P.; Hartwell, E.E. A meta-analysis of the efficacy of gabapentin for treating alcohol use disorder. *Addiction* **2019**, *114*, 1547-1555, doi:10.1111/add.14655.
24. Kristensen, S.R.; Horder, M. The influence of extracellular magnesium on cell damage induced by ATP depletion in human fibroblasts. *Scand J Clin Lab Invest* **1991**, *51*, 11-15.
25. Altura, B.M.; Barbour, R.L.; Dowd, T.L.; Wu, F.; Altura, B.T.; Gupta, R.K. Low extracellular magnesium induces intracellular free Mg deficits, ischemia, depletion of high-energy phosphates and cardiac failure in intact working rat hearts: a 31P-NMR study. *Biochim Biophys Acta* **1993**, *1182*, 329-332, doi:10.1016/0925-4439(93)90077-e.
26. Kang, T.S.; Woo, S.W.; Park, H.J.; Lee, Y.; Roh, J. Comparison of genetic polymorphisms of CYP2E1, ADH2, and ALDH2 genes involved in alcohol metabolism in Koreans and four other ethnic groups. *Journal of clinical pharmacy and therapeutics* **2009**, *34*, 225-230,

doi:10.1111/j.1365-2710.2008.00986.x.
27. Baraona, E.; Abittan, C.S.; Dohmen, K.; Moretti, M.; Pozzato, G.; Chayes, Z.W.; Schaefer, C.; Lieber, C.S. Gender differences in pharmacokinetics of alcohol. *Alcoholism, clinical and experimental research* **2001**, *25*, 502-507.
28. Zuccoli, G.; Pipitone, N. Neuroimaging findings in acute Wernicke's encephalopathy: review of the literature. *AJR Am J Roentgenol* **2009**, *192*, 501-508, doi:10.2214/AJR.07.3959.
29. Wolff, D.; Gross, M. Temporal bone findings in alcoholics. Preliminary report on chronic alcoholics. *Arch Otolaryngol* **1968**, *87*, 350-358, doi:10.1001/archotol.1968.00760060352002.
30. Muniswamy, S.; Maliakel, S.F. A Comparative Study on the Health Problems and Substance Abuse among the Tobacco Farmers and Non-Tobacco Farmers in Hassan District, Karnataka. *Indian J Occup Environ Med* **2021**, *25*, 33-38, doi:10.4103/ijoem.IJOEM_41_20.
31. Meng, H.; Zhou, Q.; Chen, S. Anti-Ma encephalitis masquerading as Wernicke encephalopathy. *J Clin Neurosci* **2020**, *79*, 160-162, doi:10.1016/j.jocn.2020.06.019.
32. Itoh, A.; Nakashima, T.; Arao, H.; Wakai, K.; Tamakoshi, A.; Kawamura, T.; Ohno, Y. Smoking and drinking habits as risk factors for hearing loss in the elderly: epidemiological study of subjects undergoing routine health checks in Aichi, Japan. *Public Health* **2001**, *115*, 192-196, doi:10.1038/sj.ph.1900765.
33. Nageris, B.I.; Ulanovski, D.; Attias, J. Magnesium treatment for sudden hearing loss. *Ann Otol Rhinol Laryngol* **2004**, *113*, 672-675, doi:10.1177/000348940411300814.
34. Curhan, S.G.; Eavey, R.; Shargorodsky, J.; Curhan, G.C. Prospective study of alcohol use and hearing loss in men. *Ear Hear* **2011**, *32*, 46-52, doi:10.1097/AUD.0b013e3181f46a2f.
35. Gomez-Casati, M.E.; Murtie, J.C.; Rio, C.; Stankovic, K.; Liberman, M.C.; Corfas, G. Nonneuronal cells regulate synapse formation in the vestibular sensory epithelium via erbB-dependent BDNF expression. *Proc Natl Acad Sci U S A* **2010**, *107*, 17005-17010, doi:10.1073/pnas.1008938107.
36. Suzuki, J.; Corfas, G.; Liberman, M.C. Round-window delivery of neurotrophin 3 regenerates cochlear synapses after acoustic overexposure. *Scientific reports* **2016**, *6*, 24907, doi:10.1038/srep24907.

37. Ernfors, P.; Van De Water, T.; Loring, J.; Jaenisch, R. Complementary roles of BDNF and NT-3 in vestibular and auditory development. *Neuron* **1995**, *14*, 1153-1164, doi:10.1016/0896-6273(95)90263-5.
38. Yu, Q.; Chang, Q.; Liu, X.; Wang, Y.; Li, H.; Gong, S.; Ye, K.; Lin, X. Protection of spiral ganglion neurons from degeneration using small-molecule TrkB receptor agonists. *J Neurosci* **2013**, *33*, 13042-13052, doi:10.1523/JNEUROSCI.0854-13.2013.
39. Shibata, S.B.; Cortez, S.R.; Beyer, L.A.; Wiler, J.A.; Di Polo, A.; Pfingst, B.E.; Raphael, Y. Transgenic BDNF induces nerve fiber regrowth into the auditory epithelium in deaf cochleae. *Exp Neurol* **2010**, *223*, 464-472, doi:10.1016/j.expneurol.2010.01.011.
40. Erol, A.; Karpyak, V.M. Sex and gender-related differences in alcohol use and its consequences: Contemporary knowledge and future research considerations. *Drug Alcohol Depend* **2015**, *156*, 1-13, doi:10.1016/j.drugalcdep.2015.08.023.
41. Aasheim, E.T. Wernicke encephalopathy after bariatric surgery: a systematic review. *Ann Surg* **2008**, *248*, 714-720, doi:10.1097/SLA.0b013e3181884308.
42. Chen, Y.; Zong, L.; Zhao, Q.; Liu, C. Advances in research on medications for the treatment of alcohol use disorders: A review. *Medicine* **2024**, *103*, e40045, doi:10.1097/MD.0000000000040045.
43. Wheeler, D.C.; Dewolfe, A.S.; Rausch, M.A. Audiometric configuration in patients being treated for alcoholism. *Drug Alcohol Depend* **1980**, *5*, 63-68, doi:10.1016/0376-8716(80)90171-4.
44. Golabek, W.; Niedzielska, G. Audiological investigation of chronic alcoholics. *Clin Otolaryngol Allied Sci* **1984**, *9*, 257-261, doi:10.1111/j.1365-2273.1984.tb01507.x.
45. Sasa, M.; Takaori, S.; Matsuoka, I.; Miyazaki, T.; Miyazaki, K. Peripheral and central vestibular disorders in alcoholics. A three-year follow-up study. *Arch Otorhinolaryngol* **1981**, *230*, 93-101, doi:10.1007/BF00665384.
46. Ribeiro, S.B.; Jacob, L.C.; Alvarenga Kde, F.; Marques, J.M.; Campelo, R.M.; Tschoeke, S.N. Auditory assessment of alcoholics in abstinence. *Braz J Otorhinolaryngol* **2007**, *73*, 452-462, doi:10.1016/s1808-8694(15)30097-5.
47. Ylikoski, J.S.; House, J.W.; Hernandez, I. Eighth nerve alcoholic neuropathy: a case report with light and electron microscopic findings. *J Laryngol Otol* **1981**, *95*, 631-642, doi:10.1017/s0022215100091209.
48. Spitzer, J.B.; Ventry, I.M. Central auditory dysfunction among chronic alcoholics. *Arch Otolaryngol* **1980**, *106*, 224-229,

doi:10.1001/archotol.1980.00790280032006.
49. Chu, N.S.; Squires, K.C.; Starr, A. Auditory brain stem responses in chronic alcoholic patients. *Electroencephalogr Clin Neurophysiol* **1982**, *54*, 418-425, doi:10.1016/0013-4694(82)90205-x.
50. Chan, Y.W.; McLeod, J.G.; Tuck, R.R.; Feary, P.A. Brain stem auditory evoked responses in chronic alcoholics. *Journal of neurology, neurosurgery, and psychiatry* **1985**, *48*, 1107-1112, doi:10.1136/jnnp.48.11.1107.
75. Page, M.J.; McKenzie, J.E.; Bossuyt, P.M.; Boutron, I.; Hoffmann, T.C.; Mulrow, C.D.; Shamseer, L.; Tetzlaff, J.M.; Akl, E.A.; Brennan, S.E.; et al. The PRISMA 2020 statement: an updated guideline for reporting systematic reviews. *Bmj* **2021**, *372*, n71, doi:10.1136/bmj.n71.
